# A population-based study of the influence of socioeconomic status on prostate cancer diagnosis in Taiwan

**DOI:** 10.1186/s12939-018-0792-2

**Published:** 2018-06-15

**Authors:** Chi-Chen Wu, Ching-Heng Lin, Han-Sun Chiang, Ming-Je Tang

**Affiliations:** 1 Graduate Institute of International Business, College of Management, National Taiwan University, No.1, Sec. 4, Roosevelt Rd, Taipei City, 106 Taiwan, Republic of China; 2 Fu Jen Catholic University, No. 510 Zhongzheng Rd., Xinzhuang Dist, New Taipei City, 24205 Taiwan, Republic of China; 3 Urological Department, Fu Jen Catholic University Hospital, No. 69, Guizi Rd., Taishan Dist, New Taipei City, 24352 Taiwan, Republic of China; 4 Department of Medical Research, Taichung Veterans General Hospital, 1650 Taiwan Boulevard Sec. 4, Taichung, 40705 Taiwan, Republic of China; 5 Department of Public Health, College of Medicine, Fu Jen Catholic University, No. 510 Zhongzheng Rd., Xinzhuang Dist, New Taipei City, 24205 Taiwan, Republic of China; 6 Department of Health Care Management, National Taipei University of Nursing and Health Sciences, No. 365, Ming-te Road, Peitou District, Taipei, 11219 Taiwan, Republic of China

**Keywords:** Socioeconomic status, Prostate cancer, Incidence rate, Stage at diagnosis, Health disparity

## Abstract

**Background:**

Disparities in prostate cancer (PCa) outcomes and their links to socioeconomic status (SES) have been intensively studied. A relatively low incidence rate and a high proportion of late-stage diagnosis have been documented in studies of Asian populations. For the past 20 years, the trend in the growth of PCa cases in Taiwan was opposite to that of Western countries. However, there is a striking paucity of local studies on these important issues. To mitigate this gap in knowledge, we exploited two population databases to investigate the impact of SES on PCa incidence rate and stage at diagnosis. Particularly, we sought to explore the discriminating capabilities of various indexes of SES on two diagnostic outcome indicators.

**Method:**

We conducted a population-based, follow-up, observational study. Data of study populations and newly diagnosed PCa cases between 2011 and 2013 were collected from the National Health Insurance Research Database and the Taiwan Cancer Registry. We retrieved 50–79 old male subjects who were classified as government employee, enterprise employee, or labor class. People with a diagnosis of any type of cancer before January 1, 2011, were excluded. The influences of four independent variables, i.e., age, beneficiary’s insurance status, occupation and income, were analyzed. We used Cox proportional hazard models to calculate the hazard ratios of PCa and used logistic regression models to analyze the odds ratios (ORs) of late-stage PCa diagnosis.

**Results:**

The low crude PCa incidence rate (112 per 100,000 person-years) and the high percentage of late-stage presentation (44%) were similar to those found in previous studies of old Asian men. Unsurprisingly, age was consistently revealed to be the most determinant factor in PCa diagnosis, while the insurance status of the beneficiaries showed no significant difference. Significant socioeconomic disparities in PCa diagnosis were demonstrated by occupation and income indexes, individually or in combination. However, occupation and income showed varied capabilities in discriminating disparities between different outcome indicators.

**Conclusion:**

Our study supported the findings of extant works showing that advantaged populations have a higher PCa incidence rate and a lower percentage of late-stage diagnosis. The discriminating capabilities of health disparity by occupation and/or income were contingent on the choice of health outcome indicators. The relatively high percentage of late-stage presentation is a critical public health challenge, and a tailored coping strategy is urgently needed. For more effective health policy-making, local socioeconomic effects on the other outcome indicators of PCa, such as incidence to mortality ratio, warrant further investigation.

## Background

A substantial number of studies on the relationship between socioeconomic status (SES) and health disparity have convinced scholars and policy makers of the significance, pervasiveness and persistence of this phenomenon [1–5]. Not all of the research findings are consistent [6, 7], but they have come to the following consensus: the poor have poorer health [5, 8]. Nevertheless, inverse relationships between SES and cancer outcomes, especially late-stage diagnosis, have been frequently documented [9, 10]. Intriguingly, that advantaged populations suffer from a higher incidence of prostate cancer (PCa) is one of the few exceptions [11, 12].

PCa has long been the most common type of male cancer in Europe and North America [6, 7]. Global PCa incidence and mortality are expected to increase to 1.7 million new cases and 499,000 PCa-specific deaths by 2030 [13]. In contrast, the National Institutes of Health projected that new PCa cases in the United States would decrease to 161,360 with 26,730 deaths in 2017 [14]. In Taiwan, the incidence of PCa has increased consistently over the past 27 years, representing the fifth most common type of male cancer in 2014 [15]. Given these discrepancies between Taiwan and developed countries, the local trends in case growth, presentation of clinical staging at diagnosis and their associations with SES warrant careful examination [16].

The authors have identified three reasons to study the relationship between SES and PCa outcomes in the specific context of Taiwan. First, SES is a complicated construct [17, 18], and studying the relationship between SES and health disparity has a long history [19, 20]. As their correlations have changed over time and space [6, 7, 18, 21], these significant socioeconomic determinants merit persistent monitoring and investigation. Second, most research in this domain has been conducted in Western countries [2, 6, 7, 12, 22–24], while investigations using data from Asian populations are relatively scarce [25]. To the best of our knowledge, only one research in Taiwan has examined the associations of PCa and SES [26]. Third, the trends in PCa incidence and stage at diagnosis in Taiwan are opposite to those of the United States. Thus, it is doubtful that local patterns for the association between SES and PCa diagnostic outcomes would be the same as those in Western countries.

To fill these knowledge gaps, we analyzed data from two population databases to answer the following questions: (1) What is the PCa incidence rate and distribution of disease stage at diagnosis (both diagnostic outcome indicators were abbreviated as PCa diagnoses) among senior males in Taiwan? (2) What are the relationships between various SES indexes and different diagnostic outcome indicators? Furthermore, (3) what are the differences between our findings and the extant evidences, and if differences exist, what are their policy implications? These answers may contribute to facilitating international comparisons, decision-making of anti-PCa policy and targeting the populations with poorer health that need to receive better care.

## Methods

### Data sources

All data were obtained from the following two population-based databases: the National Health Insurance Research Database (NHIRD) and the Taiwan Cancer Registry (TCR). These two databases can be linked by a unique encryption identity number. NHIRD was established in 1995 when the compulsory National Health Insurance (NHI) was first deployed [27]. By 2010, NHI had enrolled over 23 million citizens, representing 99% of the total population. According to NHI Act, Chapter 1 Article 2, every employed citizen must apply for the insurance by him/herself. The not-employed people can also join the insurance program through an employed close family member who must be a spouse or first linear ascendant. Thus, NHIRD defines beneficiaries by two insurance statuses: the employed insured and not-employed dependents, representing two different ways to join the NHI. Meanwhile, all beneficiaries can be classified into 15 social statuses. The insured are classified based on their occupational class, specific lifestyle or unique situation. Intriguingly, NHIRD assigns a reference status to dependents that is the same as their attached family member’s status.

The majority of beneficiaries were categorized as government employees, enterprise employees or members of the labor classes. The insured in the above occupations must be paid above minimum wage in conformance with legislative requirements, and the wage information was used to calculate individual insurance premiums. As the not-employed dependents had no wage, the wage of their attached family served as their reference income information. The other 12 statuses include employers, freelancers, soldiers, veterans, members of certain professional associations, monks and nuns, and sentenced persons. Each of the categories has its own characteristic features, and the incomes vary widely from category to category, ranging from the highest to below the poverty line. The not-employed individuals who also had no family associate to whom they could be attached were classified as a unique social status category. Accordingly, every beneficiary had a traceable social status and income information, i.e., his/her own or referenced from his/her family.

The second database, TCR, was founded by the Ministry of Health and Welfare in 1979. Hospitals with a capacity of more than 50 beds are obligated to report the detailed information of certain newly diagnosed cancers to TCR. PCa was not included in this mandatory reporting list until 2008 [28].

### Study design and study population

As a retrospective, follow-up, observational study, the study period was set from 2011 to 2013. The study populations were defined by the following eligibility criteria. All beneficiaries were first separated into two groups: the insured and the dependents. Next, we included male subjects who were actively covered on January 1, 2011 and who were aged between 50 to 79 years old. The age interval was determined based on the following considerations: (1) a large proportion of PCA cases are diagnosed in men aged more than 65 years; (2) PCa is an indolent and slow-growing disease, even though scholars have stressed that the greatest socioeconomic impacts on health occur in mid-adulthood, i.e., age 45–65 years [29]; (3) we referenced seminal researches on this topic and found that the age intervals were defined between 55 and 75 years [6, 7]; and (4) a lower incidence rate has been identified in Asian men, and the life expectancy of male Taiwanese in different counties is between 75 and 80 years.

**Fig. 1:**
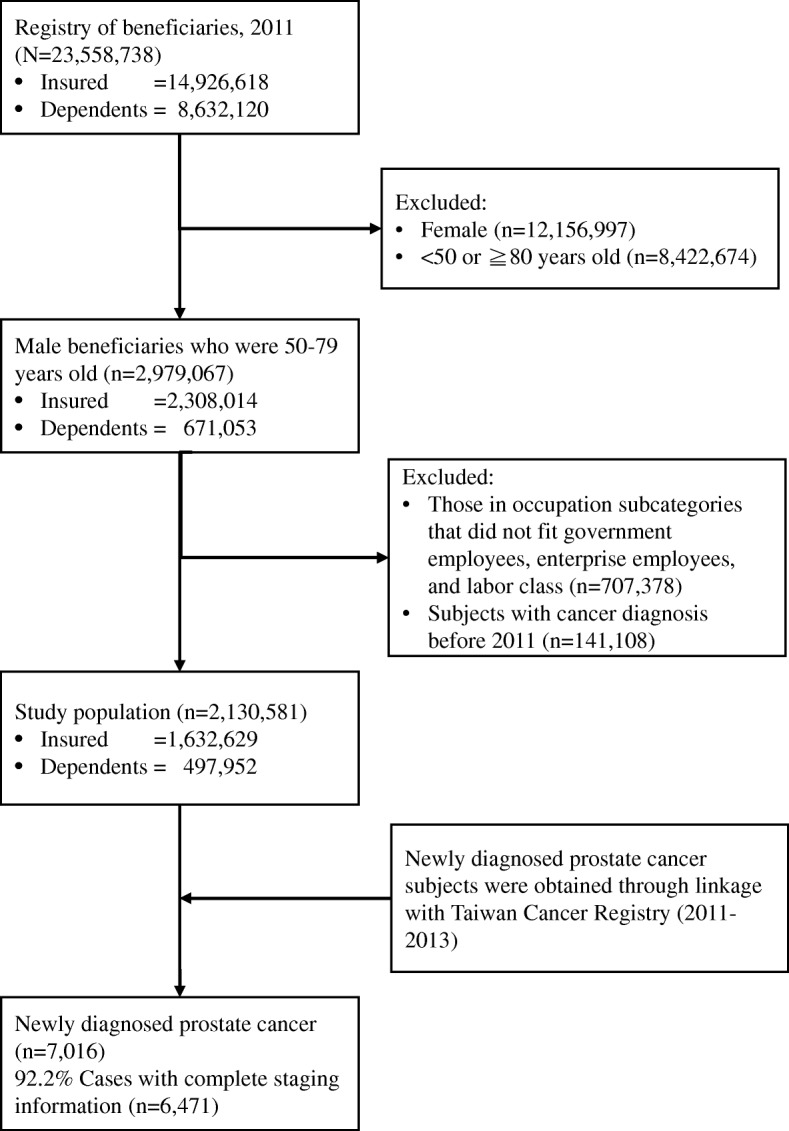
Flow chart for selection of the study population

Further, we retrieved subjects whose social statuses belonged to the categories of government employee, enterprise employee or labor class (Fig. 1), and we excluded people of the other 12 status categories. As these included subjects were all salaried employees, they must be paid above minimum wage (17,880 Taiwan Dollars (TWD) per month; approximately 600 USD). Subjects who had existing cancer diagnoses were also excluded (defined by The International Classification of Diseases, Ninth Revision, Clinical Modification (ICD-9-CM) diagnosis codes of 140.xx-208.xx). Moreover, PCa cases were collected from the TCR database. Cases with complete staging data were categorized into the early-stage group (stage I and II) or the late-stage group (stage III and IV) because of their distinct outcomes.

### Socioeconomic stratifications

There were four independent variables in this study: (1) age – the most influential factor for PCa diagnosis based on current evidence; (2) beneficiary’s insurance statuses – either the employed insured or not-employed dependents; and (3 and 4) occupation and income – two common measures used to stratify SES. The study population was grouped based on occupational classes. Government employees included civil servants and teaching staffs. Most of these individuals held undergraduate degrees or above, and had to pass strict national examinations. In general, these subjects were better paid and had more confidence in their job security. Subjects in the labor class were farmers, fishermen and so-called blue-collar workers. As a rule, these individuals received less education and had lower income and less job security. The compositions of the enterprise employee category were more diverse than those of the other two groups, and the average education level, income, and job security of enterprise employee were expected to lie between those of labor class and government employee.

The statutory retirement age is 65 years, although the actual average retirement age is around 63 years for male citizens in Taiwan. However, the majority of labor workers continue to keep their jobs into their seventies. In general, Taiwanese government employees are recognized as more advantaged than enterprise employees, while the laborers are considered the least advantaged in the hierarchy.

As employers are required to report employees’ monthly wages, we decided to use this accurate and complete information to represent income status. In NHIRD, wage data are divided into fifty-four levels with ten different intervals. To avoid data manipulation, we used quantiles of the total population wage data to divide the study population into high-, middle- and low-income levels. Accordingly, high income was defined as a monthly wage of more than 40,100 TWD (the exchange rate was 30 TWD to one US Dollar); middle income as between 21,901 and 40,100 TWD; and low income as between 17,880 TWD (the minimum wage) and 21,900 TWD. As the income distribution was highly positively skewed, the subjects were concentrated in the low-income area, which resulted in more people in the low-income group than those in the other two groups (approximately 4:3:3).

In this paper, we used the insurance status of the beneficiary as the third categorical variable. As mentioned previously, the key difference between the beneficiaries was employment status, which has been recognized as a potential source of socioeconomic difference [30, 31]. We exploited this special arrangement to study whether there were health gradients within the not-employed population and, if there were, whether the patterns of health disparities of the dependents were the same as those of the insured.

### Data analysis

We first applied a chi-square test to show the characteristic compositions of the insured group versus the dependent group. Next, we calculated the crude incidence rate and the percentage of late-stage diagnosis separately. After that, we subdivided each group into three age intervals and recalculated the ratios to show the age-adjusted effects.

Furthermore, we conducted four separate univariate analyses to test the individual influences of age, insurance status, occupation and income on PCa diagnoses. At the same time, we performed multivariable analyses in four models to illustrate their discriminating effects. In Model 1, we added the age variable to the insurance-status univariate analysis. Next, the occupation variable was added to Model 1 to create Model 2. For Model 3, we replaced the occupation variable of Model 2 with the income variable. In the full model, Model 4, all the independent variables were simultaneously analyzed. Using Cox proportional hazard model analysis, we analyzed the effects of the four variables on the PCa incidence rate. To find the influences of the independent variables on the odds ratio (OR) of late-stage presentation, we ran logistic regression analyses in the same sequence as described above.

We executed database cleaning, merging, and analysis using SAS, version 9.4 (SAS Institute Inc., Cary, NC). All analyses were two-tailed, and the level of significance was 0.05. All data were retrieved and processed within the Data Science Center of the Ministry of Health and Welfare, Taiwan.

## Results

As shown in Fig. 1, following the occupational status inclusion criteria, we retrieved 1,632,629 subjects as the insured group and 497,952 subjects as the dependents group. The total of 2,130,581 subjects accounted for 72% of the 50 to 79 year-old male population. According to the composition analysis by insurance statuses, there were statistically significant differences (all *p* < 0.001, Table 1) in the distributions of age, occupation and income between the insured and the dependent groups, indicating that these two groups of people cannot automatically be recognized as one population sharing the same disparities before further analysis, especially when the assigned reference status of the dependents was referenced from the attached family.

**Table 1:** Characteristics of the study population by beneficiary’s insurance status (*n* = 2,130,581)

Variables	Insured (*n* = 1,632,629)		Dependents (*n* = 497,952)		*p*-value
	Number	Percent	Number	Percent	
Age, yrs					< 0.001
50–59	1,074,346	65.8	141,016	28.3	
60–69	365,487	22.4	219,037	44.0	
70–79	192,796	11.8	137,899	27.7	
Occupation					< 0.001
Labor	943,053	57.8	166,377	33.4	
Enterprise employee	592,942	36.3	288,350	57.9	
Government employee	96,634	5.9	43,225	8.7	
Income, TWD					< 0.001
17,881–21,900	687,511	42.1	176,375	35.4	
21,901–40,100	422,743	25.9	192,608	38.7	
> 40,100	522,375	32.0	128,969	25.9	

By linking the NHIRD and TCR databases, we identified 7016 newly diagnosed PCa cases that were used to calculate the incidence rate. Of these, 6471 cases (approximately 92%) had complete staging information, and only these data were used in the OR analysis of late-stage presentation. No significant difference (*p* = 0.75) was found between two insurance statuses.

### PCa diagnoses of the senior population in Taiwan

**Fig. 2:**
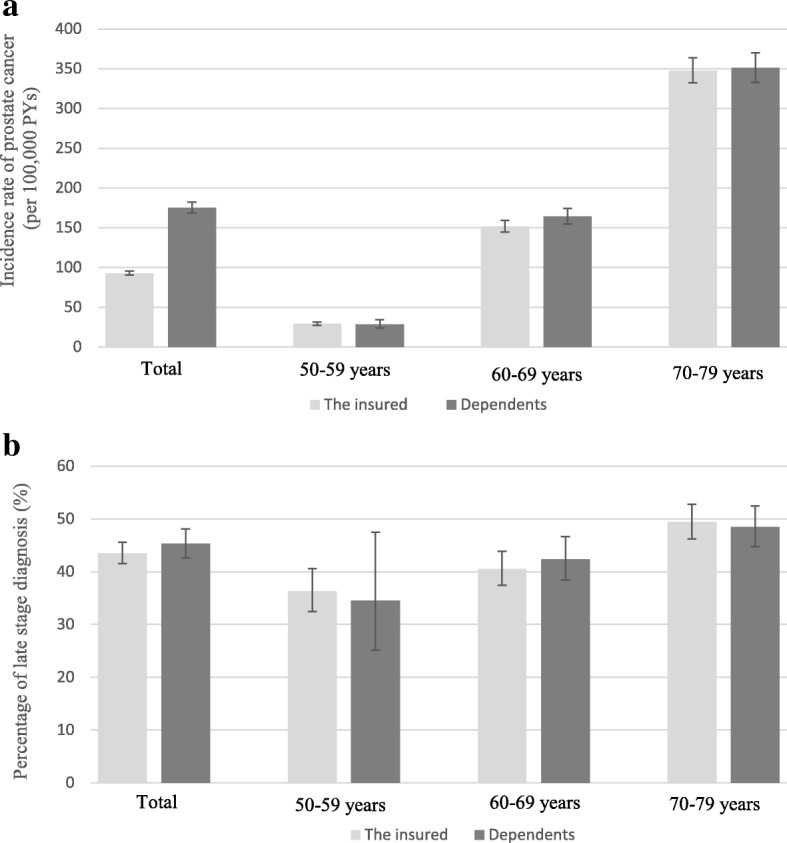
The PCa incidence rate of the study population (**a**) and the percentage of late-stage presentation of PCa cases (**b**) by insurance statuses and age

In this study, we demonstrated that the crude incidence rate of PCa in 50 to 79 year-old males was 112/100,000 person-years (PYs), and approximately 44% of PCa cases were confirmed as late stage at diagnosis. Interestingly, the difference in the crude incidence rate between the insured and dependents was absent in the following three age interval comparisons (Fig. 2a). This absence was due to the combined influences of age on PCa diagnosis and the large differences in the age distribution between these two groups (Table 1). However, age did not show such dramatic effects on the proportion of cases with late-stage diagnosis. Nevertheless, a higher ratio of late-stage presentation in dependent group could be identified in Fig. 2b.

### SES and PCa diagnoses

In the univariate analyses, all four variables demonstrated significant disparities in PCa diagnoses (all *p* < 0.01, in Table 2 and Table 3) except for the insurance status in the OR analysis (univariate model in Table 3). Unsurprisingly, age was evidenced to be the most significant risk factor of PCa [32]. The incidence rate of the 70 to 79 year-old group was around 12 times higher than that of the 50 to 59 year-old group (HR: 12.02, 95% CI: 11.22–12.89, *p* < 0.001), and the discriminating capability persisted throughout all analyses. Compared with the 50 to 59-year-old group, the 70 to 79 year-old group had a 70% higher tendency for late-stage presentation, and the 60 to 69 year-old group had a 24% higher tendency for late-stage presentation. The beneficiary’s insurance status predicted a false-positive significant difference in incidence rate, as explained above. There was no significant difference found in late-stage presentation between the insured and dependents. However, the unexpected trends in the intergroup health gradients demonstrated by the occupation and income indexes required further analysis.

**Table 2:** Univariate and multivariable Cox proportional hazard models of prostate cancer incidence

Variables	Univariate Models			Model 1			Model 2			Model 3			Model 4		
	HR	(95% CI)	*p*-value	HR	(95% CI)	*p*-value	HR	(95% CI)	*p*-value	HR	(95% CI)	*p*-value	HR	(95% CI)	*p*-value
Group															
Insured	1.00	(reference)		1.00	(reference)		1.00	(reference)		1.00	(reference)		1.00	(reference)	
Dependents	1.89	(1.80–1.98)	< 0.001	1.04	(0.99–1.09)	0.149	0.96	(0.90–1.01)	0.118	1.01	(0.96–1.07)	0.711	0.98	(0.93–1.04)	0.515
Age, yrs															
50–59	1.00	(reference)		1.00	(reference)		1.00	(reference)		1.00	(reference)		1.00	(reference)	
60–69	5.38	(5.01–5.77)	< 0.001	5.32	(4.95–5.72)	< 0.001	5.51	(5.12–5.92)	< 0.001	5.47	(5.08–5.89)	< 0.001	5.48	(5.09–5.90)	< 0.001
70–79	12.02	(11.22–12.89)	< 0.001	11.89	(11.08–12.77)	< 0.001	12.61	(11.71–13.58)	< 0.001	12.43	(11.52–13.41)	< 0.001	12.44	(11.53–13.42)	< 0.001
Occupation															
Labor	1.00	(reference)		–	–	–	1.00	(reference)		–	–	–	1.00	(reference)	
Enterprise employee	0.81	(0.76–0.86)	< 0.001	–	–	–	1.17	(1.10–1.24)	< 0.001	–	–	–	1.14	(1.05–1.23)	0.002
Government employee	1.13	(1.03–1.23)	0.008	–	–	–	1.33	(1.21–1.46)	< 0.001	–	–	–	1.23	(1.10–1.39)	< 0.001
Income, TWD															
17,881–21,900	1.00	(reference)		–	–	–	–	–	–	1.00	(reference)		1.00	(reference)	
21,901–40,100	0.55	(0.51–0.58)	< 0.001	–	–	–	–	–	–	0.96	(0.90–1.03)	0.212	0.89	(0.82–0.96)	0.003
> 40,100	0.72	(0.68–0.76)	< 0.001	–	–	–	–	–	–	1.25	(1.18–1.33)	< 0.001	1.11	(1.02–1.21)	0.019

Models 1–4 are multivariable Cox proportional hazard models

**Table 3:** Univariate and multivariable logistic regression models of late-stage diagnosis in patients with prostate cancer

Variables	Univariate Models			Model 1			Model 2			Model 3			Model 4		
	OR	(95% CI)	*p*-value	OR	(95% CI)	*p*-value	OR	(95% CI)	*p*-value	OR	(95% CI)	*p*-value	OR	(95% CI)	*p*-value
Group															
Insured	1.00	(reference)		1.00	(reference)		1.00	(reference)		1.00	(reference)		1.00	(reference)	
Dependents	1.08	(0.97–1.19)	0.159	1.01	(0.91–1.12)	0.917	1.13	(1.01–1.27)	0.034	1.11	(0.99–1.24)	0.076	1.12	(1.00–1.26)	0.060
Age, yrs															
50–59	1.00	(reference)		1.00	(reference)		1.00	(reference)		1.00	(reference)		1.00	(reference)	
60–69	1.24	(1.07–1.45)	0.006	1.24	(1.06–1.45)	0.007	1.15	(0.98–1.36)	0.079	1.10	(0.94–1.30)	0.246	1.11	(0.94–1.30)	0.226
70–79	1.70	(1.47–1.98)	< 0.001	1.70	(1.46–1.98)	< 0.001	1.54	(1.31–1.80)	< 0.001	1.43	(1.21–1.69)	< 0.001	1.45	(1.23–1.71)	< 0.001
Occupation															
Labor	1.00	(reference)		–	–	–	1.00	(reference)		–	–	–	1.00	(reference)	
Enterprise employee	0.78	(0.70–0.87)	< 0.001	–	–	–	0.81	(0.72–0.91)	< 0.001	–	–	–	0.99	(0.83–1.17)	0.902
Government employee	0.62	(0.51–0.75)	< 0.001	–	–	–	0.61	(0.50–0.74)	< 0.001	–	–	–	0.80	(0.62–1.03)	0.079
Income, TWD															
17,881–21,900	1.00	(reference)		–	–	–	–	–	–	1.00	(reference)		1.00	(reference)	
21,901–40,100	0.82	(0.72–0.93)	0.002	–	–	–	–	–	–	0.85	(0.74–0.98)	0.027	0.87	(0.73–1.04)	0.132
> 40,100	0.63	(0.56–0.71)	< 0.001	–	–	–	–	–	–	0.67	(0.59–0.76)	< 0.001	0.71	(0.58–0.85)	< 0.001

Models 1–4 are all multivariable logistic regression models

Multivariable analyses showed that insurance status lost its explanatory power (all *p* > 0.05) in predicting disparities of PCa diagnoses except in Model 2 of Table 3 (*p* = 0.34) when analyzed together with age and occupation. Occupation and income revealed significant gradients in PCa diagnoses when analyzed with age and insurance status variables (Model 2 and Model 3 of Table 2 and Table 3).

In full model analyses, both occupation and income predicted that the advantaged subjects had higher incidence rates (all *p* < 0.05, Model 4 of Table 2). Unexpectedly, the middle-income group was found to have the lowest incidence rate (HR: 0.89, 95% CI: 0.82–0.96, *p* = 0.003), a finding that called for further elaboration. On the other hand, only the highest-income group demonstrated a significantly lower ratio of late-stage presentation (OR: 0.71, 95% CI: 0.58–0.85, *p* < 0.001). The other occupation and income indexes did not show significant intergroup disparities in late-stage presentation, as displayed in Model 4 of Table 3.

## Discussion

Based on our study design, authors showed that the relationship between SES and PCa diagnosis was compatible with extant evidences. As expected, age was dominant in predicting PCa risk, while beneficiary’s insurance status did not show significant differences in most of our analyses. Occupation and income indexes did not consistently demonstrate the socioeconomic disparities in PCa diagnoses in the same way, especially when used in combination. These findings will be discussed in detail below.

### PCa in Taiwan

Our findings showed that, essentially, the more advantaged population had a higher PCa incidence rate and lower late-stage presentation ratio [22, 23, 25, 33]. However, the crude incidence rate of 112/100,000 PYs in our high-risk, senior study population was much lower than those found in Europe and the US [1, 2]. Similarly, an official report of the age-standardized PCa incidence rate, which was derived from the same databases in 2014, showed 29.1 per 100,000 PYs [34]. As to the staging statistics, in this population, 44% of PCa cases were late-stage presentations, while 56% showed an early stage at diagnosis, which is much lower than the current 80 to 92% rate of localized, early-stage diagnosis in the US [14, 35]. This information might be valuable for clinical professionals and policy makers, especially for those focusing on the Han Chinese population [36]. Additionally, these findings indicate that PCa is a critical and urgent public health challenge in Taiwan.

Some contextual information needs to be explained before interpreting and generalizing our results. The main characteristics of the health care system in Taiwan are that NHI is a mandatory, single-payer insurance system [37], and almost all hospitals have joined the system to serve this intensively competitive and highly efficient medical care market [27]. However, there has been no large-scale PCa screening trial in Taiwan. One possible explanation is that PCa was not one of the top five causes of male cancer until recently. When PCa is suspected, either incidentally through self-paid health examinations or symptomatically evidenced, patients only pay approximately 570 TWD (less than 20 USD) for a urological outpatient appointment and they can go directly to a tertiary medical center without a gatekeeper’s referral. Necessary diagnostic examinations and conventional therapies are comprehensively covered by NHI, while, unfortunately, no annotation had been made about why beneficiaries sought medical assistances in NHIRD. However, this does not mean that there is no barrier for the less-advantaged population to receive adequate medical care.

### SES measures and health outcome indicators

Occupation and income indexes are among the most commonly used measures in health disparity research [31, 38]. The basic assumptions suggest that higher incomes provide people with better nutrition, housing, schooling, and means to purchase health care resources. Also, more privileged occupations carry less risks of excessive job strain, occupational injury, exposures to toxic substances, and so forth. Both of which make the advantaged population healthier [38]. By and large, the advantaged are recognized as having a lower cancer incidence rate except for some frequently overdiagnosed cancers, such as PCa, breast cancer and thyroid cancer [39]. Researchers also identify stage at diagnosis as a complementary but important indicator to determine the phenomenon of overdiagnosis [40]. That is, when the advantaged suffer from a higher cancer incidence rate, they might simultaneously have the benefit of fewer late-stage presentations at diagnosis, if they are not experiencing complications resulting from overtreatment [41]. Consequently, the incidence rate combined with the stage at diagnosis must be examined together when performing cancer epidemiological research [24, 30, 39].

### The role of the beneficiary’s insurance status

We did not find a significant difference in PCa diagnoses between the employed insured and not-employed dependents in this study. The main reason for this finding could be the choice of targeted disease. In addition, the health impacts of the dependent’s insurance status cannot fully represent the health effects of unemployment, which consist of voluntary and involuntary unemployed causes [42]. Indeed, at least half of our study population was older than the average retirement age, 62.8 years, for males in Taiwan (statutory retire age: 65 years), and there was no annotation for why dependents were not-employed. Furthermore, Shavers emphasized that the difficulties associated with classifying retired and unemployed subjects might lead to inconsistent results [31].

Notably, even though no differences were found between the insurance statuses in the incidence rate analysis, there was an exceptional finding of a statistically significant difference in late-stage diagnosis in Model 2 of Table 3 (*p* = 0.034, OR: 1.13, 95% CI: 1.01–1.27) and two borderline differences in Model 3 (*p* = 0.076) and Model 4 (*p* = 0.06). Although the differing effects were not remarkable in our study, we propose that the influence of the insurance status warrants further investigation, especially in younger populations and properly selected disease targets.

### The role of occupation

In this study, occupation showed significant discriminating capability in most of our analyses except in the full model (Model 4), as shown in Table 3. Hence, we conducted a Spearman’s correlation test of the four independent variables. The results highlighted that, on average, the advantaged occupation groups were younger and better paid, and a higher percentage of them were identified as dependents (correlation coefficient: − 0.122, 0.68, and 0.19, respectively; all *p* < 0.0001; *n* = 2,130,581). These correlations offered some explanation as to why occupation lost its differentiating capability, but the explanations were also outcome-indicator dependent. Our results support the arguments of Alder and Ostrove [43] stating that when more than one SES indicator was used, health outcomes might be correlated with one indicator more than the other.

Each group of researchers has its own preferences in measure selection, although most tend not to offer reasons for why their respective measures were chosen [17]. In Great Britain, occupation has been routinely collected in all official datasets and surveys [38] and was commonly used in seminal health inequality researches [44, 45]. While the status-list of NHIRD was originated from a mix of legacy professional insurance programs, it did not follow the occupation classification principles or standards of Western countries. Nevertheless, the individual occupational classes here represented an average and socially recognized level of education, income, job security and assessment of health care resources.

### The role of income

The majority of empirical findings support the assertion that more affluent populations have higher PCa incidence rates. Unexpectedly, we identified an enhanced intergroup disparity of the middle-income group, which showed the lowest incidence rate (HR = 0.89, 95% CI = 0.82–0.96, *p* < 0.003; Model 4 of Table 2). There are three probable explanations for this finding. First, epidemiological transition may have occurred, which means that the trends in socioeconomic disparities in health might have changed along with societal changes and economic developments [21, 46]. Second, disparities in PCa diagnoses is an exceptional case, especially when case growth had reached a plateau during study period. These suggestions could be verified by performing a similar study using other cancer types or through longitudinal approaches. Third, the result may be influenced by certain lifestyle factors among the local middle-income population that gives them special protection, such as green tea intake [47]. Although these hypotheses require further evaluation, our findings indicate that status syndrome [48, 49] may not apply to all scenarios.

### Reflections and implications

#### Weaknesses and limitations

We did not examine the entire Taiwanese population due to the following concerns: 1) The excluded 28 % of the targeted population (Fig. 1) belonged to the other 12 social status categories, and each of these 12 categories contained far fewer members than the three occupational classes. 2) Subjects of certain social statuses live a distinct lifestyle or in a unique situation that might influence the discovery or development of PCa. These subjects include sentenced persons, monks and nuns, and statutory low-income households. However, these special populations warrant further investigation in the future. 3) Because we sought to explore the possible differences in socioeconomic disparities in health due to measure selection, each subject needed to have traceable information about his/her social status and income information. Therefore, we confined our study population to three major classes of people.

Moreover, we did not use wage criteria (income) as inclusion criteria for three reasons. First, in NHIRD, wage data are divided into fifty-four levels with ten different intervals, and each level includes a different number of people. Second, the distribution of income among all beneficiaries was highly positively skewed. Third, all beneficiaries were classified into 15 social status categories, which means that each wage level might comprise people of different social statuses, not to mention fluctuations in the number of people in each wage level. Hence, if we used wage criteria for inclusion, then there would have been a varied mix of social status categories and significant fluctuation in the number of people in different income quantiles, and this approach would have made the analyses too complicated to interpret.

The rationale of using the SES of the attached employed family member to serve as the reference status of the not-employed dependents was inspired from the socioeconomic disparities of children’s academic performance, where the parent’s SES was used as the reference status [50]. Given that background, the drawback is that it is not possible to rule out the potential blunting or exaggerating of effects of the reference status on the exposed health disparities of the dependent population. To explore the potential distortion effects, education index should be a logical alternative because every beneficiary has their own level. As there is no education information in NHIRD, this represents a weakness of this study. In addition, the application of self-developed aggregated occupation classes and monthly wages may not be standard practices of SES measurement [17, 31]. However, the accuracy and completeness of the data source [27, 28] make these approaches trustworthy. In addition, as there is no information about why dependents were not employed, we lost the opportunity to examine the potential health impacts of unemployment.

To generalize our findings, we also need to focus on contextual differences between studies. For instance, our study materials came from a population-based insurance database, which is a different population from those of randomized PCa screening studies [6, 7], and the characteristics of the insurance system and the intense competition of the medical service market in Taiwan should be taken into account as well [37].

#### Strengths and contributions

Most studies select only one SES measure because of data availability or multicollinearity considerations [17]. Here, we apply one insurance status variable and two SES indexes, which offered us an opportunity to observe the individual and combined effects on different outcome indicators [17, 51]. Through these efforts, this research contribute the first Taiwanese study on socioeconomic disparities of PCa diagnosis and provide further support to the interregional differences between Asian and Western countries [16, 25].

#### Suggestions for future studies

Although we have identified the poorest of the poor population who should be served first, we still need a tailored strategy to cope with our unique epidemiological presentations of PCa, i.e., the low absolute incidence rate, high ratio of late-stage presentation and opposite trends in case growth compared to those of developed countries. To provide solid foundations for effective coping strategies, longitudinal approaches of local studies about the relationships between SES and comprehensive health outcome indicators, e.g., health knowledge, choice of therapeutic modalities, prognosis, complications, and mortality to incidence ratios, are highly expected.

Based on our findings, some knowledge gaps still remain. First, does the lowest incidence rate found in the middle-income group occur only in PCa, or is it a common phenomenon for all cancers in Taiwan? Next, as SES is a dynamic construct, the health impacts of social mobility need to be clarified. Third, the increasing trends in incidence rate of PCa in Taiwan indicate that the knowledge diffusion of preventive and therapeutic guidelines for PCa may also differ from those in Western countries. It warrants further investigation. Fourth, as we understand more about the social determinants of PCa diagnoses, we must emphasize the significance of developing better diagnostic tools than the currently used PSA test [52]. Not until we can detect PCa earlier and predict PCa progress more precisely, the troublesome consequences of overdiagnosis and overtreatment can be effectively managed.

## Conclusion

The incidence rate and late-stage diagnosis ratio of PCa in Taiwan are correlated with individual SES, and the high percentage of late-stage presentation stands out as a critical public health challenge for local government. Either the occupation or income index is suitable for demonstrating socioeconomic gradients of PCa diagnoses, but the efficacy of these indexes will not be as good when used in combination. Finally, because of the unique trends in PCa incidence, unacceptably high proportion of late-stage presentation, as well as evidenced racial protection effects, we suggest that a tailored anti-PCa strategy is desperately needed. Given the accelerating aging and increasing social inequality in Taiwan, closing the health gap is becoming more and more challenging.

## Abbreviations

ICD-9-CM: The International Classification of Diseases, Ninth Revision, Clinical Modification
NHI: National Health Insurance
NHIRD: National Health Insurance Research Database
OR: Odds Ratio
PCa: Prostate Cancer
PSA: Prostate-Specific Antigen
PYs: Person-Years
SES: Socioeconomic Status
TCR: Taiwan Cancer Registry
TWD: Taiwan New Dollar
USD: United States Dollar
